# Leaf gas exchange and water relations of the woody desiccation-tolerant *Paraboea rufescens* during dehydration and rehydration

**DOI:** 10.1093/aobpla/plac033

**Published:** 2022-07-31

**Authors:** Pei-Li Fu, Ya Zhang, Yong-Jiang Zhang, Patrick M Finnegan, Shi-Jian Yang, Ze-Xin Fan

**Affiliations:** CAS Key Laboratory of Tropical Forest Ecology, Xishuangbanna Tropical Botanical Garden, Chinese Academy of Sciences, Menglun, Mengla, Yunnan 666303, China; Ailaoshan Station of Subtropical Forest Ecosystem Studies, Xishuangbanna Tropical Botanical Garden, Chinese Academy of Sciences, Jingdong, Yunnan 676209, China; Anhui Provincial Key Laboratory of the Conservation and Exploitation of Biological Resources, College of Life Sciences, Anhui Normal University, Wuhu, Anhui 241000, China; School of Biology and Ecology, University of Maine, Orono, ME 04469, USA; School of Biological Sciences, University of Western Australia, 35 Stirling Highway, Perth, WA 6009, Australia; School of Ecology and Environmental Science, Yunnan University, Kunming, Yunnan 650500, China; CAS Key Laboratory of Tropical Forest Ecology, Xishuangbanna Tropical Botanical Garden, Chinese Academy of Sciences, Menglun, Mengla, Yunnan 666303, China; Ailaoshan Station of Subtropical Forest Ecosystem Studies, Xishuangbanna Tropical Botanical Garden, Chinese Academy of Sciences, Jingdong, Yunnan 676209, China; Center of Plant Ecology, Core Botanical Gardens, Chinese Academy of Sciences, Guangzhou, Guangdong 510650, China

**Keywords:** Desiccation tolerance, Gesneriaceae, leaf hydraulic conductance, *Paraboea rufescens*, rehydration, resurrection plant, root pressure

## Abstract

Desiccation-tolerant (DT) plants can withstand dehydration to less than 0.1 g H_2_O g^−1^ dry weight. The mechanism for whole-plant recovery from severe dehydration is still not clear, especially for woody DT plants. In the present study, we evaluated the desiccation tolerance and mechanism of recovery for a potentially new woody resurrection plant *Paraboea rufescens* (Gesneriaceae). We monitored the leaf water status, leaf gas exchange, chlorophyll fluorescence and root pressure of potted *P. rufescens* during dehydration and rehydration, and we investigated the water content and chlorophyll fluorescence of *P. rufescens* leaves in the field during the dry season. After re-watering from a severely dehydrated state, leaf maximum quantum yield of photosystem II of *P. rufescens* quickly recovered to well-watered levels. Leaf water status and leaf hydraulic conductance quickly recovered to well-watered levels after re-watering, while leaf gas exchange traits also trended to recovery, but at a slower rate. The maximum root pressure in rehydrated *P. rufescens* was more than twice in well-watered plants. Our study identified *P. rufescens* as a new DT woody plant. The whole-plant recovery of *P. rufescens* from extreme dehydration is potentially associated with an increase of root pressure after rehydration. These findings provide insights into the mechanisms of recovery of DT plants from dehydration.

## Teaser

Desiccation-tolerant (DT) plants can survive in extremely dry environments and withstand dehydration to less than 0.1 g H_2_O g^−1^dry mass, and after rehydration the DT plants will regain normal function. This study provides the first evidence that *Paraboea rufescens*, a small shrub from Gesneriaceae, is a DT plant. After re-watering from a severely dehydrated state, leaf water status and leaf physiological function of *P. rufescens*can quickly recover to the well-watered levels. This study also shows that root pressure is an important driving force for whole-plant recovery in *P. rufescens*from severe desiccation.

## Introduction

Desiccation-tolerant (DT) plants can survive in extremely dry environments and withstand dehydration to less than 0.1 g H_2_O g^−1^ dry weight (DW). This equilibrates to 50 % relative air humidity and corresponds to a leaf water potential (Ψ_leaf_) of about −100 MPa (Gaff 1971; Alpert 2006). Such plants regain normal function after rehydration. Desiccation tolerance is a common phenomenon in lichens, mosses and ferns (Alpert 2006). It is also present in the seeds of most angiosperm species (Liu *et al.* 2008). However, desiccation tolerance is rare in the vegetative tissues of angiosperm species. Only about 135 angiosperm species have been reported to have desiccation tolerance (Gaff and Oliver 2013). These DT plants can be divided into two types, i.e. homoiochlorophyllous and poikilochlorophyllous (Lüttge *et al.* 2011). Homoiochlorophyllous retain their light-absorbing pigment complexes during dehydration, while poikilochlorophyllous lose their photosynthetic apparatus and thus avoid photodamage (Tuba *et al.* 1998).

One challenge for DT vascular plants during the rehydration process is that they need to refill their xylem after complete embolism during dehydration. The two possible mechanisms that could contribute to xylem refilling in vascular DT plants are capillary rise and positive root pressure. Sherwin *et al.* (1998) suggested that capillary rise was the main driving force for xylem refilling in *Myrothamnus flabellifolia*, which is the only known angiosperm DT plant with a woody stem (Moore *et al.* 2007). However, Schneider *et al.* (2000) argued that root pressure, not capillary pressure, was the main driving force for xylem refilling of this species. Root pressure was also found to be an important driving force for xylem refilling of two terrestrial DT ferns (Holmlund *et al.* 2020). However, it is unknown if root pressure is a common mechanism for recovery from complete dehydration in angiosperm DT plants.

Most DT angiosperms are short. That is because the DT plant must remove completely xylem embolism after re-watering from the severely dry state. For example, the height of the tallest woody DT angiosperm plant *M. flabellifolia* is up to 0.8 m (Gaff and Oliver 2013). Desiccation-tolerant plants from Velloziaceae were shorter and showed greater decreases in photosynthetic rate during the dry season than the non-DT species (Alcantara *et al.* 2015). Moreover, clarifying the response of leaf gas exchange and hydraulics in DT plants to dehydration is important for understanding the adaptive strategies of plants to survive in extreme drought conditions.

Gesneriaceae is a large family with 133 genera and more than 3000 species distributed in tropical and subtropical regions (Wang *et al.* 1998). Many species from this family are endemic and endangered, because of their limited distribution area and habitat (Wiehler 1983). Nine genera within the Gesneriaceae have been reported to contain DT species (Gaff and Oliver 2013). Lüttge *et al.* (2011) proposed that there could be DT plants in the genus *Paraboea* (Gesneriaceae); however, there is no direct evidence to support this proposal.

**Figure 1.**
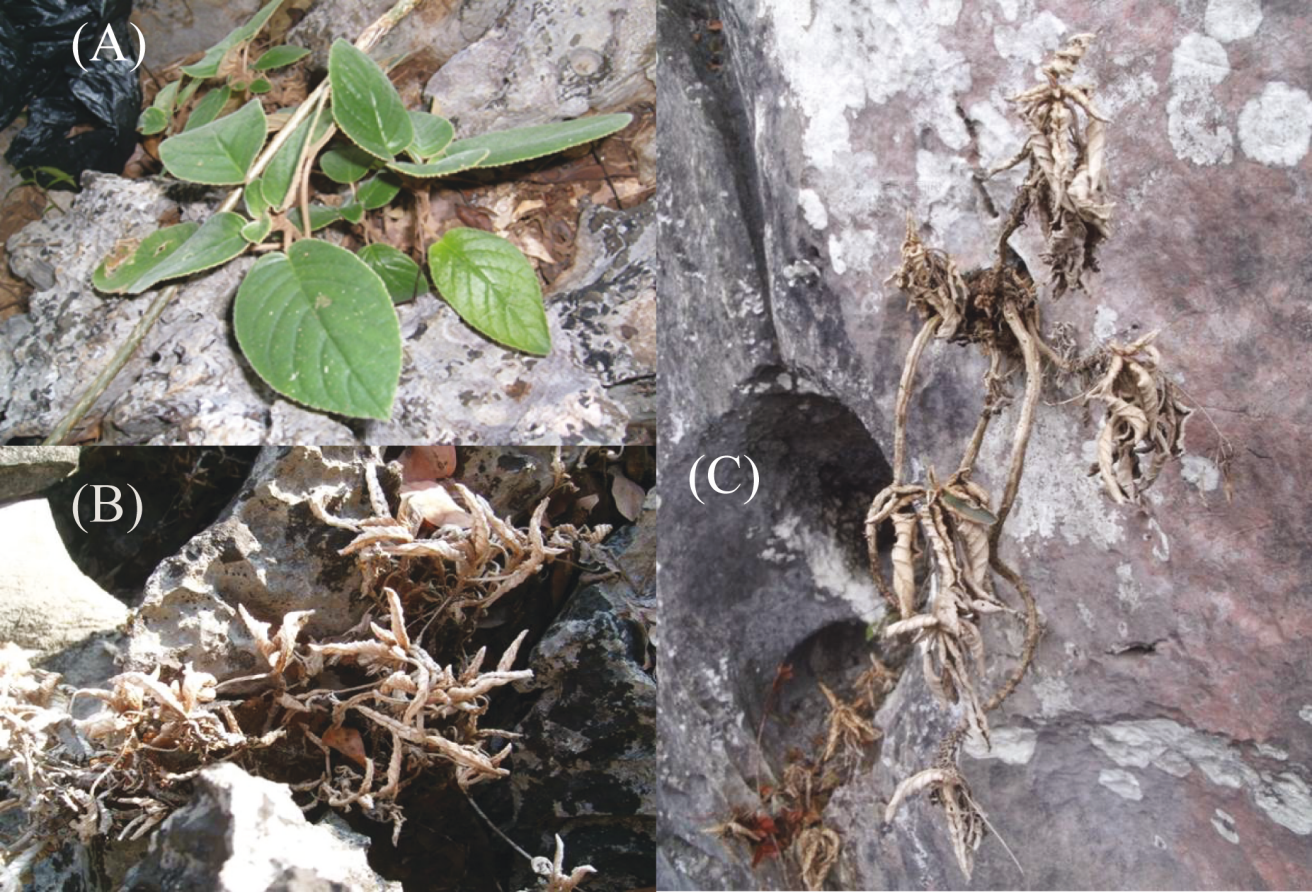
Photographs of well-watered and dehydrated *Paraboea rufescens* in the field. (A) A well-watered *P. rufescens* in the rainy season. (B) A dehydrated *P. rufescens* in the dry season. (C) A dehydrated *P. rufescens* with a long stem in the dry season.

*Paraboea rufescens* is widely distributed in the karst habitats of southwest China (Wang *et al.* 1998). This species is abundant at the top of karst hills, where water availability can be severely limited in the dry season. Previous studies on this species include reproductive biology (Gao *et al.* 2006) and leaf chlorophyll fluorescence (Huang *et al.* 2012). Although *P. rufescens* was referred as a ‘resurrection plant’ by Huang *et al.* (2012), no direct evidence was provided. *Paraboea rufescens* has a woody stem that can be longer than 40 cm, with leaves clustering near the top of the stem (Fig. 1). In the dry season, leaves of *P. rufescens* curl severely and turn their abaxial side outward; however, they unfold and recover completely in the rainy season or after a rain in the dry season. Such characteristics of *P. rufescens* are similar to DT plants. However, there are no reports demonstrating the desiccation tolerance of this species. We hypothesized that (i) *P. rufescens* was a DT plant, able to recover the photosynthetic apparatus from severe dehydration and (ii) after re-watering, root pressure will increase above the value of hydrostatic pressure corresponding to the plant height, which may play an important role in the whole-plant recovery from severe dehydration. We monitored the leaf water status, leaf gas exchange, chlorophyll fluorescence and root pressure of potted *P. rufescens* during dehydration and rehydration, and we investigated the water content and chlorophyll fluorescence of *P. rufescens* leaves in the field during the dry season.

## Materials and Methods

### Study site and plant material

The study site is located at a karst hill (21°55ʹN, 101°15ʹE, 700 m a.s.l.) in Xishuangbanna, Yunnan, China. This region has a monsoon climate with a distinct dry season from November to April. The annual mean precipitation is 1500 mm, and the annual mean temperature is 21 °C. More than 80 % of the rainfall occurs in the rainy season from May to October.

Severely dehydrated leaf and stem samples were collected from each of nine *P. rufescens* individuals in the field during the peak of the dry season in 2016. The water content of these leaves and stems was measured. Two leaves from a similar position on each individual were sampled. One leaf was used to measure leaf water content. The other leaf was rehydrated by putting the leaf onto a filter paper wet with water in a Petri dish for 24 h, as described by Deng *et al.* (2003). Maximum quantum yield of photosystem II (PS II) (*F*_v_/*F*_m_) was measured for the rehydrated leaves.

Twenty *P. rufescens* individuals were collected from the top of the karst hill in October 2014 and planted separately into pots (10 cm in diameter × 10 cm depth) for dehydration and rehydration experiments. The potted individuals were put in a shade house with the light level reduced to 50 % by a shade cloth. The shade house was rain-proofed with a plastic roof cover. Plants were well-watered and kept in the shade house for 2 months before water was withheld, and five individuals were well-watered throughout the experiment. During the dehydration process, the mean temperature was 15.5 °C and mean relative humidity was 89 %. The mean light intensity of the shade house was 185 μmol m^−2^ s^−1^. Dehydration was ended when the leaf water content was lower than 0.10 g H_2_O g^−1^ DW, which took 27 days. Plants were then re-watered at 20:00 by adding water alone to the soil in the pots, and measurements were conducted at about 10:00 of the next day, which was 14 h after re-watering. Measurements on the rehydration plants were repeated on five continuous days at about the same time. During both dehydration and rehydration processes, leaf water content, water potential, hydraulic conductance, gas exchange rate and quantum yield of photosynthetic system II (Φ_PSII_) were monitored. Maximum quantum yield of PS II was also measured for well-watered individuals and re-watered individuals on the third, fourth and fifth days after re-watering. Five leaves from each of five individuals were selected for the above measurements. Rehydration was ended when the leaf water content of the re-watered individuals reached the same level as the well-watered control individuals.

### Leaf and stem water content

Leaves and 50-mm length of stems with bark removed were weighed to determine fresh weight of samples collected from the karst hill, and then dried at 70 °C for 48 h to determine DW. Leaf and stem water content were expressed as g H_2_O g^−1^ DW. Leaf water content was measured similarly during the dehydration and rehydration process. The relative water content (RWC, %) was calculated by dividing the absolute water content by the mean value of the saturated leaf water content from the leaf pressure–volume curve, which was 2.69 g H_2_O g^−1^ DW.

### Maximum quantum yield of PS II

Leaf maximum quantum yield of PS II (*F*_v_/*F*_m_) was measured for the field-collected leaves after rehydration, well-watered potted individuals and rehydrated individuals on the third, fourth and fifth day of rehydration with a chlorophyll fluorescence meter (FMS2, Hansatech, Norfolk, UK). Dark-adapting clips affixed to leaves for at least 1 h before measurement. The minimum fluorescence (*F*_o_) was measured firstly, and then the maximum fluorescence (*F*_m_) was measured after a saturating light pulse of 5000 μmol m^–2^ s^–1^. The *F*_v_/*F*_m_ was calculated as (*F*_m_ − *F*_o_)/*F*_m_ (Maxwell and Johnson 2000).

### Leaf pressure–volume curve

Five stems from each of five *P. rufescens* individuals were collected from well-watered potted plants. Stem segments were re-cut at the base and put into water to rehydrate for 2 h. A leaf pressure–volume curve was constructed using a bench-drying method (Turner 1988). Leaf water potential was measured with a pressure chamber (PMS 1000, Corvallis, OR, USA). Leaf weight and positive pressure inside the chamber were measured periodically until the pressure developed in the chamber stopped increasing. The leaves were then dried at 70 °C for 48 h to determine the DW. Prior to evaluation, the curve was visually inspected for indications of excess apoplastic water (plateau effect), which may be an artefact that sometimes accompanies leaf rehydration (Kubiske and Abrams 1991; **see****Supporting Information—Method S1**). Turgor loss point water potential (Ψ_tlp_, MPa), osmotic potential at full turgor (π_100_, MPa), leaf water content at turgor loss point, bulk modulus of elasticity along the entire range of positive turgor pressure (ε, MPa), and saturated leaf water content (g H_2_O g^−1^ DW) were then calculated from the pressure–volume curve **[see****Supporting Information—Method S1**, Fig. S4**]**.

### Leaf gas exchange and quantum yield of photosynthetic system II

Light-saturated area-based photosynthetic rate (*A*_a_, μmol m^−2^ s^−1^), maximum stomatal conductance (*g*_s_, mol m^−2^ s^−1^) and quantum efficiency of photosynthetic system II (Φ_PSII_) were measured between 9:00 and 11:00 with a portable leaf gas exchange system embedded with a chlorophyll fluorescence head (LI-6400, Li-COR, Lincoln, NE, USA). The conditions inside the leaf chamber were 400 ppm CO_2_ at 25 °C and 50 % relative humidity, with a photosynthetic photon flux density (PPFD) of 1000 μmol m^–2^ s^–1^. Leaves were equilibrated in the chamber condition for at least 5 min before the leaf gas exchange rate was recorded. Steady-state fluorescence yield (*F*_t_) was measured, followed by the maximum fluorescence in the light (*F*'ʹ_m_) after a saturating light pulse of 8000 μmol m^–2^ s^–1^ light. Φ_PSII_ was calculated as (*Fʹ*_m_ − *F*_t_)/*Fʹ*_m_ (Maxwell and Johnson 2000). *A*_a_ and *g*_s_ were measured until the 10th day of dehydration, while Φ_PSII_ was measured until the 12th day of dehydration. After 12 days, the leaves were severely curled, preventing further measurements.

### Leaf water potential

Leaf water potential (Ψ_leaf_, MPa) was measured with a pressure chamber immediately after leaf gas exchange measurements. Leaf water potential was measured until the 13th day of dehydration. Severe shrinkage of the leaf blade and petiole prevented further measurements.

### Leaf hydraulic conductance

Leaf hydraulic conductance was measured using an evaporative flux method (Sack *et al.* 2002). An excised leaf was immediately connected to a pipette via a water-filled tube. The leaf was fixed horizontally onto an open web made with fishing line above a fan and under full sunlight to drive transpiration. The transpiration flow rate (*E*, mmol s^−1^) was measured when a steady *E* was reached. The leaf was then transferred into a moist zip-lock plastic bag. Leaf water potential was measured with a pressure chamber after at least 30 min equilibration within the leaf. The leaf area (LA, cm^2^) was measured with a leaf area meter (LI-3000A, Li-COR). Leaf area-based hydraulic conductance (*K*_leaf_, mmol m^−2^ s^−1^ MPa^−1^) was calculated as *E*/(−Ψ_leaf_ × LA). *K*_leaf_ was measured until the 13th day of withholding water. Severe shrinkage of the leaf blade and petiole prevented further measurements.

### Root pressure measurement

Root pressure of four well-watered *P. rufescens* individuals was monitored with digital pressure transducers (PX26-100DV, Omega Engineering, Stamford, CT, USA) following the procedure of Cao *et al.* (2012). A stem from each individual was cut 2 cm above the ground in the evening. The cut end of the remaining potted plant was immediately connected to a pressure transducer via a water-filled tube. Root pressure was logged every 5 min (CR1000 datalogger, Campbell Scientific, Logan, UT, USA) for 2 days. The value of root pressure of well-watered individuals decreased to negative values and bubbles started to appear in the tube because of daytime transpiration. The tubes were refilled with water and reconnected to the cut end of the stem on the second evening. In addition, root pressure for four individuals from the water-withholding experiment was monitored similarly at the end of the dehydration phase. After re-watering, the pressure transducer was installed immediately to the cut end of the stem on each potted individual. Root pressure data were again logged every 5 min until rehydration ended. Bubbles started to appear in the tubes at the morning of the second day after re-watering (36 h after re-watering). The tubes were refilled with water and reconnected to the cut end of the stem each evening. Root pressure of the re-watered plants was monitored until the sixth day after re-watering.

### Statistical analyses

The differences among means across days in leaf water content, Ψ_leaf_, *K*_leaf_, *g*_s_, *A*_a_, Φ_PSII_ and *F*_v_/*F*_m_ during the dehydration and re-watering were compared using Tukey’s honest significant difference test. The relationships between Ψ_leaf_ and *g*_s_, *A*_a_, *K*_leaf_ and Φ_PSII_ during dehydration and re-watering were fitted using a generalized additive mode (‘gam’, Hastie 2020). All data analyses were done in R ver. 4.0.2 (R Core Team 2020)

## Results

### Water status and maximum quantum yield (*F*_v_/*F*_m_) of PS II for *P. rufescens*

**Figure 2.**
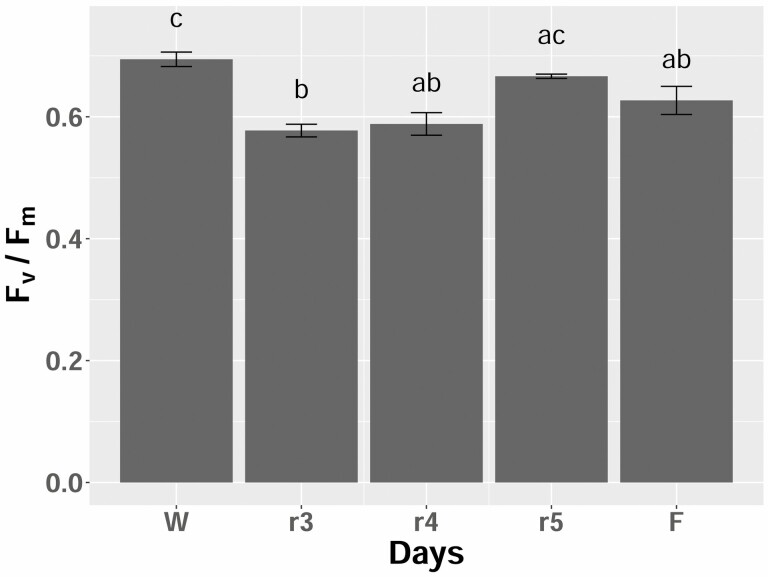
Leaf dark-adapted maximum quantum yield of photosynthetic system II (*F*_v_/*F*_m_) of re-watered and well-watered *Paraboea rufescens*. Means ± SE (*n* = 4–5) for leaves from well-watered plants (W), for leaves from severely dehydrated potted plants after re-watering for 3 (r3), 4 (r4) and 5 days (r5) and for rehydrated leaves collected from the field at the peak of the dry season (F, *n* = 9) are shown. Different letters above the bars indicate significant differences among means across treatments compared using Tukey’s honest significant difference test (*P <* 0.05).

**Figure 3.**
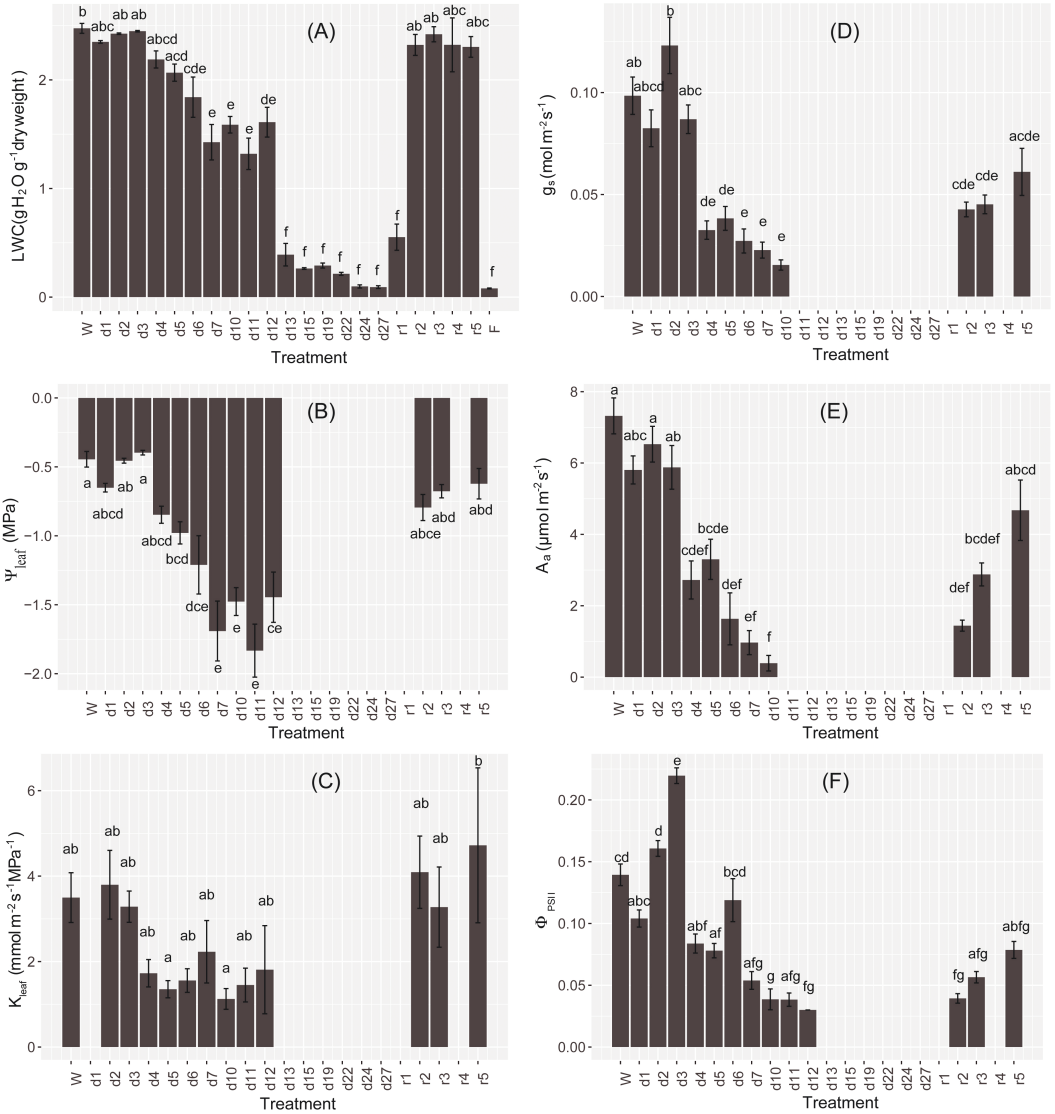
Changes in leaf water status, gas exchange and chlorophyll fluorescence in *Paraboea rufescens* during dehydration and re-watering. (A) leaf water content (LWC), (B) leaf water potential (Ψ_leaf_), (C) leaf hydraulic conductance (*K*_leaf_), (D) maximum stomatal conductance (*g*_s_), (E) leaf area-based photosynthetic rate (*A*_a_) and (F) quantum efficiency of photosynthetic system II (Φ_PSII_). Values were means ± SE (*n* = 4–5) taken from well-watered individuals (W), after withholding water for 1–27 days (d1–d27) and after re-watering for 1–5 days (r1–r5). The LWC of leaves collected from the field at the peak of dry season is also given (‘F’ in panel A, *n* = 9). Different letters above the bars indicate significant differences among means across treatment compared using Tukey’s honest significant difference test (*P* < 0.05).

The water content of leaf and stem samples of *P. rufescens* collected from the field during the peak of the dry season were 0.079 ± 0.004 g H_2_O g^−1^ DW (mean ± SE) and 0.062 ± 0.006 g H_2_O g^−1^ DW, respectively. The corresponding RWC for leaf and stem were 3.0 ± 0.1 % and 2.3 ± 0.2 %, respectively. After rehydration by foliar water uptake for 24 h, leaves unfolded completely and *F*_v_/*F*_m_ was 0.63 ± 0.02, which was 90 % of the *F*_v_/*F*_m_ in well-watered potted control plants (Fig. 2; **see****Supporting Information—Fig. S1**). In a pot experiment, after 27 days of dehydration, leaf water content of *P. rufescens* was 0.092 ± 0.006 g H_2_O g^−1^ DW (RWC 3.4 ± 0.2 %), which was similar to that of leaves collected from the field at the peak of the dry season (Fig. 3A). At this point, plants were re-watered. On the third day of re-watering of dehydrated individuals, the leaf *F*_v_/*F*_m_ value was lower than that of the well-watered control plants. However, on the fifth day of re-watering, the *F*_v_/*F*_m_ values were no longer significantly different from the well-watered control plants (Fig. 2).

### Leaf pressure–volume curve analysis for *P. rufescens*

The water potential at leaf turgor loss was −0.88 ± 0.06 MPa, and the osmotic potential at saturated turgor was −0.62 ± 0.03 MPa. The relative leaf water content at leaf turgor loss point was 78.9 ± 3.3 %, and the bulk modulus of elasticity along the entire range of positive turgor pressure was 3.42 ± 0.75 MPa. The saturated leaf water content was 2.69 ± 0.13 g H_2_O g^−1^ DW.

### Effects of dehydration and rehydration on leaf water status, gas exchange and quantum yield of PS II in *P. rufescens*

Leaf water content of potted individuals during dehydration decreased in a stepwise manner. During the first 5 days of dehydration, leaf water content was similar to that of leaves from well-watered control plants, which was 2.47 ± 0.05 g H_2_O g^−1^ DW (RWC: 92 %). Upon further dehydration, leaf water content decreased stepwise to about 1.5 g H_2_O g^−1^ DW from Day 6 to 12, and there was a dramatic drop that eventually reached below 0.1 g H_2_O g^−1^ DW on Day 27 of dehydration (Fig. 3A; **see****Supporting Information—Fig. S2**). Despite this extreme level of dehydration, leaf water content increased quickly after re-watering. The RWC was 20.5 % after 14 h (first day) of re-watering, and reached 86.4 % after 38 h (second day) of re-watering **[see****Supporting Information—Fig. S2****]**. This high RWC remained stable for the final 3 days of the experiment **[see****Supporting Information—Fig. S2****]**.

During dehydration, Ψ_leaf_ dropped gradually (Fig. 3B). By Day 4 of withholding water, Ψ_leaf_ had decreased to −0.85 ± 0.06 MPa, which was similar to the leaf turgor loss point water potential (Ψ_tlp_, −0.88 MPa) (*P* = 0.615, *t*-test). By Day 7 of dehydration, Ψ_leaf_ decreased to −1.69 ± 0.22 MPa, which was significantly lower than the Ψ_tlp_ (*P* = 0.020, *t*-test). While there was a variation among individuals and slight fluctuations among days, Ψ_leaf_ remained stable from Day 7 until Day 12 of dehydration. After the Day 12, Ψ_leaf_ could not be measured due to the strong shrinkage of the leaves. After re-watering, Ψ_leaf_ increased significantly. After re-watering for 38 h (Day 2) there was no significant difference in Ψ_leaf_ between leaves from rehydrated and well-watered control plants (Fig. 3B). After re-watering for 14 h (Day 1), leaves were still curled and folded, which prevented measurement of Ψ_leaf_, *g*_s_, *A*_a_, *K*_leaf_ and Φ_PSII_ on that day.

The *K*_leaf_ was 3.50 ± 0.58 mmol m^−2^ s^−1^ MPa^−1^ in the well-watered control plants, which decreased to 49 % by Day 4 of withholding water (Fig. 3C). However, *K*_leaf_ did not decrease further and remained ca. 50 % of the well-watered control plants until the Day 12 of dehydration. After re-watering, *K*_leaf_ also recovered quickly (Fig. 3C). Although there was some variation among individuals, *K*_leaf_ recovered to the well-watered control level after 38 h (Day 2) of re-watering and even increased to 135 % of that in leaves of well-watered plants by Day 5 of re-watering.

With the decrease in leaf water content and Ψ_leaf_, leaf stomatal conductance (*g*_s_), leaf area-based photosynthetic rate (*A*_a_) and quantum yield of photosynthetic system II (Φ_PSII_) showed a stepwise drop from Day 3 to Day 4 of dehydration (Fig. 3B, 3D–F). Upon dehydration, *g*_s_, *A*_a_ and Φ_PSII_ decreased to about 60 % and 33 % of that of well-watered plants on Day 5 and Day 10 of dehydration, respectively. Interestingly, Φ_PSII_ increased to 158 % of the value in the well-watered plants by Day 3 of dehydration. After re-watering, *g*_s_, *A*_a_, Φ_PSII_ increased incrementally, and reached 34 %, 20 % and 28 % of the values in well-watered control plants after 38 h of re-watering (Day 2), respectively. On Day 5 after re-watering, *g*_s_, *A*_a_ and Φ_PSII_ were about 60 % of the values in well-watered control plants.

### Correlation of Ψ_leaf_ with *g*_s_, *A*_a_, *K*_leaf_ and Φ_PSII_ during dehydration and rehydration

**Figure 4.**
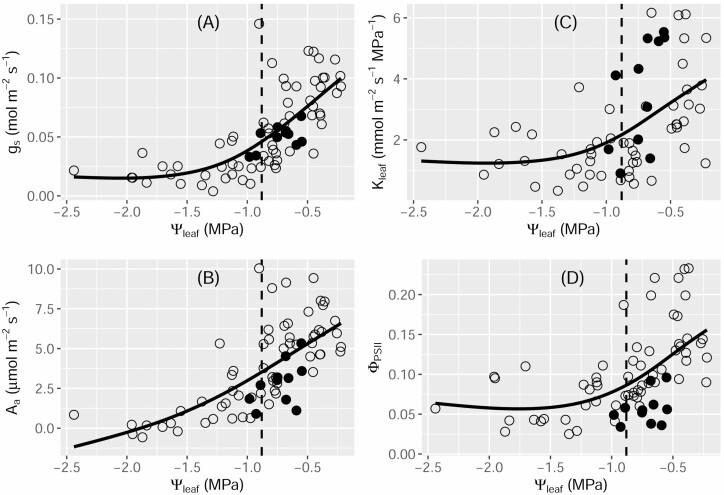
Correlations in *Paraboea rufescens* between leaf water potential (Ψ_leaf_) and stomatal conductance (*g*_s_) (A), maximum area-based photosynthetic rate (*A*_a_) (B), leaf hydraulic conductance (*K*_leaf_) (C) and quantum efficiency of photosynthetic system II (Φ_PSII_) (D). Data obtained during dehydration (open circles) and during re-watering (closed circles) are shown. Dashed lines indicate the leaf water potential at the turgor loss point (Ψ_tlp_). Black lines show the trends in the combined data from both plant dehydration and re-watering. Data were fit using the generalized additive model.

Stomatal conductance and *A*_a_ decreased dramatically with decreasing Ψ_leaf_ (Fig. 4A and B). However, the rate of the decline in *g*_s_ decreased when Ψ_leaf_ was below Ψ_tlp_. The correlation of Ψ_leaf_ with both *K*_leaf_ and Φ_PSII_ showed a similar trend to that of *g*_s_ versus Ψ_leaf_ (Fig. 4C and D), i.e. the rate of decline for both *K*_leaf_ and Φ_PSII_ decreased when Ψ_leaf_ was below Ψ_tlp_.

### Root pressure in well-watered and re-watered individuals of *P. rufescens*

**Figure 5.**
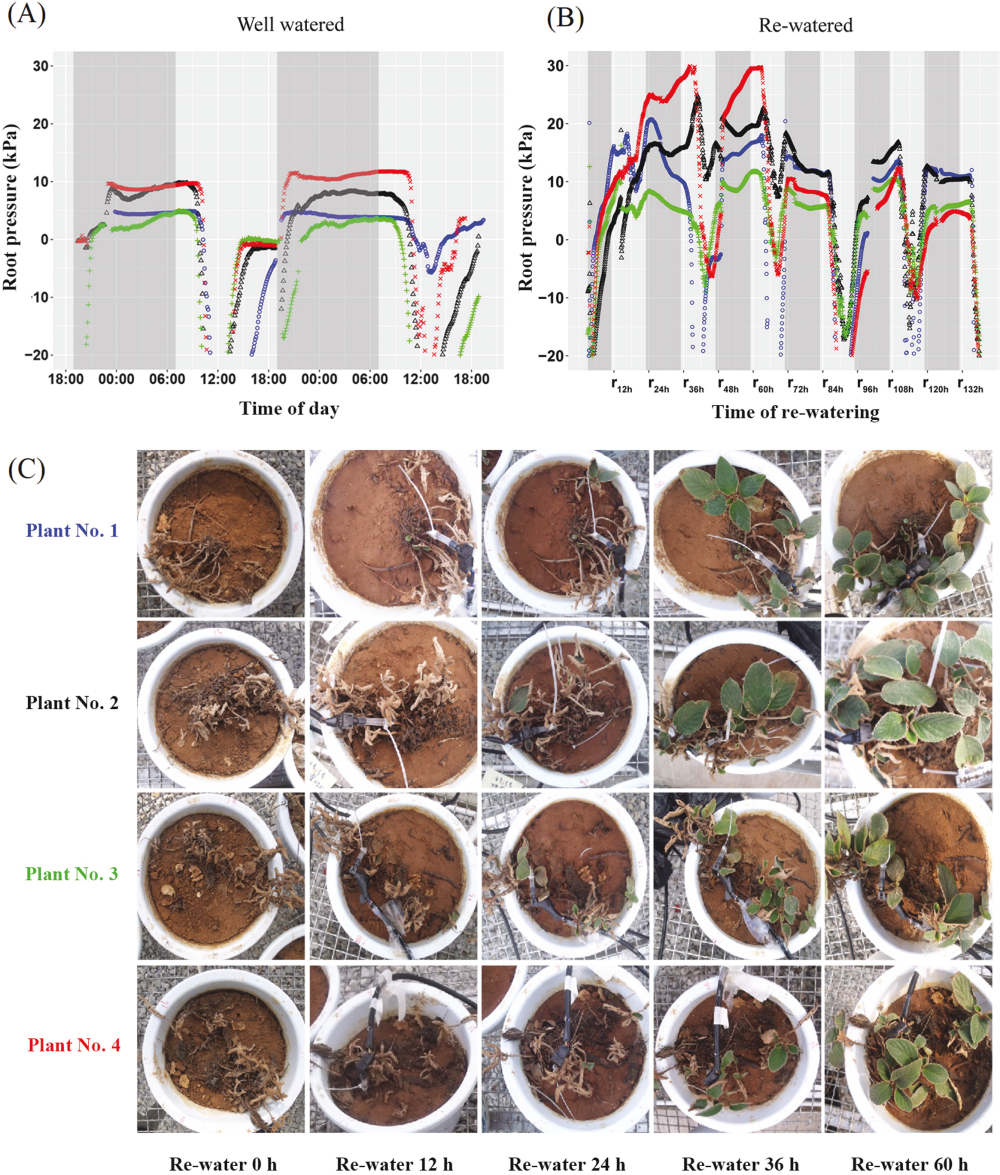
Dynamics of root pressure fluctuations in *Paraboea rufescens* in well-watered individuals (A) and in re-watered individuals recovering from severe dehydration (B), with photos of the four individuals examined during re-watering (C). Different coloured lines and symbols correspond to different individuals. The shaded areas in (A) and (B) indicate the hours of darkness from 19:00 to 7:00.

The root pressure of four well-watered *P. rufescens* individuals exposed to 50 % sunlight remained positive and constant from evening until about 9:00 of the next day. Root pressure then decreased to negative values in the middle of the day due to transpiration (Fig. 5A). The diurnal pattern of root pressure in the four well-watered individuals showed similar trends over two consecutive days. The maximum root pressure of four well-watered individuals ranged from 4.9 to 11.8 kPa, with a mean value of 7.8 ± 1.7 kPa (Fig. 5A).

Root pressure was measured in a different set of four plants that had been subjected to dehydration and then re-watered (Fig. 5B). In these plants, the diurnal pattern of root pressure fluctuation was similar to that of well-watered individuals on Day 2 of re-watering. Root pressure during the initial phase of rehydration increased continually within 24 h from the start of re-watering. The maximum root pressure during rehydration was generally higher than in well-water individuals, ranging from 11.5 to 30.4 kPa, with a mean value of 19.8 ± 4.0 kPa (Fig. 5B). The time from re-watering to reach the maximum root pressure was different among the four plants, which were 24 h, 40 h, 42 h and 60 h for plant no. 1 to no. 4, respectively (Fig. 5B). After this initial maximum, the peak root pressure decreased from day to day for the four plants. After re-watering for 72 h, the maximum root pressure approached levels similar to those in the well-watered plants. In addition, the root pressure in all four individuals was positive across most of the daylight hours on the first day of re-watering. However, during daylight hours on subsequent days, the root pressure decreased strongly and could become quite negative (i.e. −20 kPa). Within 24 h of re-watering, parts of the top leaf of all four individuals started to unfold (Fig. 5C). Most leaves at lower position on the four individuals unfolded and showed recovery after re-watering for 36 h. After re-watering for 60 h, all leaves had recovered completely.

## Discussion

### *Paraboea rufescens* is a woody DT plant

Our study provides the first evidence that *P. rufescens* is a DT plant. The leaf water content both in the field and in the dehydration experiment decreased to less than 0.1 g H_2_O g^−1^ DW without plant mortality (Fig. 3A). After re-watering from the severely dehydrated state, the maximum quantum yield of PS II (*F*_v_/*F*_m_) of detached leaves from the field and leaves of potted individuals both recovered to the levels in well-watered plants (Fig. 2). The recovery of leaf *F*_v_/*F*_m_ after rehydration is important evidence for attributing desiccation tolerance to a plant species (López-Pozo *et al.* 2019). Thus, our study confirmed the speculation of Lüttge *et al.* (2011), where the genus *Paraboea* (Gesneriaceae) was proposed to contain DT plants.

The dehydration and rehydration experiment further confirmed desiccation tolerance of *P. rufescens* (Fig. 3). Leaf water content decreased to less than 0.1 g H_2_O g^−1^ DW at the end of dehydration. However, when water was re-supplied, leaf water status and leaf hydraulic conductance increased quickly, and recovered to the well-watered level within 38 h after re-watering (Fig. 3A–C). Leaf gas exchange and chlorophyll fluorescence traits also trended to recovery, but at a slower rate than that of leaf water status and hydraulic conductance (Fig. 3D–F). Our study suggested that leaf water status and hydraulic conductance of the DT *P. rufescens* could get quickly recovered after rehydration, but the completely recovery of leaf gas exchange might take a longer time.

Although the turgor loss point water potential (Ψ_tlp_) usually corresponds with the closure of stomata so that plants avoid further dehydration (Bartlett *et al.* 2016), *A*_a_ and *g*_s_ in *P. rufescens* were still one-third of the levels in well-watered plants on Day 4 of dehydration when Ψ_tlp_ was reached (Fig. 3D and E). Thus, *P. rufescens* could maintain leaf gas exchange even after the turgor loss point. The delay of stomatal closure with dehydration would allow *P. rufescens* to continue carbon assimilation even during water stress. With further dehydration, *A*_a_ and *g*_s_ decreasing to nearly zero by the Day 10 of withholding water. After re-watering, leaf photosynthetic activity and stomatal conductance recovered gradually. Although *A*_a_ and *g*_s_ recovered to only 60 % of the well-watered individuals by Day 5 of re-watering, the dark-adapted maximum chlorophyll fluorescence (*F*_v_/*F*_m_) of rehydrated plants on this day was similar to that of well-watered plants (Fig. 2). This observation indicated that the primary photosynthetic process related to the reaction centres of PS II tended to recover faster than the following primary and secondary photosynthetic processes of enzymatic CO_2_ assimilation (Fig. 3E and F).

### The increase of root pressure after rehydration of *P. rufescens*

We found that the root pressure of rehydrated individuals of *P. rufescens* continually increased during the first 2 days after re-watering (Fig. 5), which could be potentially important for the whole-plant recovery of *P. rufescens* from severe desiccation. Root pressure in rehydrated plants ranged from 11.5 to 30.4 kPa, which was high enough to drive water to a height of 1.17 to 3.10 m, whereas the height the re-watered individuals were less than 0.4 m. High root pressure can successfully refill xylem embolisms and aid the full recovery of two DT ferns (Holmlund *et al.* 2020) and DT *M. flabellifolia* (Schneider *et al.* 2000). Interestingly, the maximum root pressure in rehydrated *P. rufescens* was more than twice that in well-watered plants that were not subjected to desiccation. This large difference in root pressure was similar to that in rice, where re-watered individuals had a root pressure that was 7-fold greater than in well-watered plants before drought (Stiller *et al.* 2003).

Root pressure of re-watered *P. rufescens* plants continued to increase until the leaves were fully unfolded. Once the leaves were unfolded, the root pressure began to decrease during daylight hours due to leaf transpiration (Fig. 5). The increase in root pressure after re-watering might be related to the high ion and solute concentrations that accumulated during the dehydration process. In addition, the expression of aquaporins during rehydration process might help to speed up the rehydration process. Since the rapid change in water permeability of root over the short term can be mostly accounted by the changes in cell membrane permeability mediated by aquaporins (Javot and Maurel 2002). Besides, Kurowska *et al.* (2019) also found that drought stress and re-watering affected the abundance of aquaporins transcript in barley. Stiller *et al.* (2003) argued that since high root pressure in rice could be maintained for 7 days after re-watering, the increased root pressure could not be caused simply by the passive accumulation of ions. However, the detailed mechanism that generates the high root pressure seen in *P. rufescens* after re-watering of severely dehydrated plants needs further investigation. Understanding the mechanism underlying increased root pressure during re-watering is important for revealing the mechanisms involved in xylem refilling in DT plants.

Capillary force has been confirmed to be an important mechanism for recovery from desiccation in *M. flabellifolia* (Sherwin *et al.* 1998). Our study could not exclude a contribution made by capillary forces to xylem refilling of *P. rufescens* after re-watering. However, root pressure after re-watering was high enough to push water well beyond the top leaves of *P. rufescens*. Guttation was also observed from leaf scars of re-watered *P. rufescens* on the very first day of re-watering **[see****Supporting Information—Fig. S3B****]**, further indicating that root pressure was in excess of that needed to push water to the top of the plant. Schneider *et al.* (2000) concluded that root pressure rather than capillary pressure was the dominant driving force for the rehydration of *M. flabellifolia*. Holmlund *et al.* (2020) also found that capillary force was insufficient to resurrect distal pinnules in two fern species, perhaps due to reduced xylem transverse area in ferns that limits the resurrection rate. Due to the hydrophobic properties of the inner xylem surface (Zimmermann *et al.* 2004), the contact angle between water and inner xylem surface in dry stem is high, which considerably limits water rise in dry stems of *M. flabellifolia* under capillary pressure (Schneider *et al.* 2000). The capillary force can resurrect proximal pinnules in the DT fern *Pellaea andromedifolia*; however, root pressure is needed for the recovery of distal pinnules (Holmund *et al.* 2020). However, studies including simultaneous monitoring of root pressure and capillary force are needed to determine the role of capillary force in the rehydration of DT plants after severe dehydration.

Foliar water uptake also played an important role in the recovery of resurrection plants from severely dehydration. The epiphytic DT fern *Pleopeltis polypodioides* could rehydrate the leaf mesophyll and recovered carbon assimilation from foliar water uptake (Prats and Brodersen 2021). In support of the importance of foliar water uptake in plant rehydration, our results showed that placing severely dehydrated leaves from the field on wet filter paper for 24 h allowed the leaves to completely unfold and the dark-adapted *F*_v_/*F*_m_ reached 90 % of that observed in well-watered plants. Foliar water uptake could be important for the leave of *P. rufescens* to get quickly recovered and back to normal function from severely dehydration after short-term rainfall events in the field. However, the whole-plant recovery would be more depended on the generation of root pressure, since the recovery of the whole plant would be critical for long-term survival and growth of new leaves for *P. rufescens*.

### The ecological significance of *P. rufescens* being a resurrection plant

Being a DT plant is an important adaptive strategy for *P. rufescens*. The annual precipitation in Xishuangbanna is about 1500 mm, of which only 16 % occurs in the dry season from November to April. Due to the low water retention capacity in a karst habitat, plants will experience periodic water deficit, even in the rainy season (Liu *et al.* 2011). The available soil water in a karst habitat after a heavy rainfall is only sufficient to meet plant transpiration demand for 7–14 days (Li *et al.* 2008). Since the distribution of *P. rufescens* is restricted to the top of karst hills, it will periodically experience extreme water deficit. According to our measurements, the water content in both leaf and stem in the dry season was below 0.1 g H_2_O g^−1^ DW. Leaf water status and hydraulic conductance in *P. rufescens* recovered on the second day after re-watering from severe dehydration (Fig. 3A–C). However, with additional foliar water uptake, the recovery of DT plants can be faster (Holmund *et al.* 2020). The leaf gas exchange rate for well-watered *P. rufescens* was higher than in *Haberlea rhodopensis* and *Ramonda myconi*, two other resurrection species from the Gesneriaceae, but was lower than that of *Craterostigma plantagineum* and two DT species from Velloziaceae (Nadal *et al.* 2021). The photosynthetic rate of *P. rufescens* was within the range of tree species growing in the same karst habitat (Fu *et al.* 2012). Desiccation-tolerant plants have greater chloroplast exposure to intercellular spaces; therefore, they may possess even higher photosynthetic rate than their desiccation-sensitive counterparts when water is not limited (Nadal *et al.* 2021). Thus, *P. rufescens* has a moderate photosynthetic rate when the water is available, whereas in the dry season, the plant can maintain some level of photosynthesis as Ψ_leaf_ drops below Ψ_tlp_, and then becomes dormant to avoid damage as it becomes more dehydrated.

Our study showed that *P. rufescens* is a woody DT plant. Leaf water status and hydraulic conductance recovered within 2 days; however, leaf gas exchange could not be recovered to the well-watered status after re-watering for 5 days. The desiccation tolerance trait is likely to be an important adaptive mechanism for *P. rufescens* to survive in the seasonally droughted environment at the tops of karst hills. Our results also showed that root pressure was significantly increased after re-watering of *P. rufescens* from extreme dehydration, which could be important for the whole-plant recovery.

## Supporting Information

The following additional information is available in the online version of this article—

**Method S1.** Data evaluation process of leaf pressure–volume curve.

**Figure S1.** Photographs of dehydrated *Paraboea rufescens* leaves collected from the field (A) and the same leaves after rehydration on wet filter paper for 24 h (B).

**Figure S2.** Changes in leaf relative water content (RWC) in *Paraboea rufescens* during dehydration and re-watering. Values are means ± SE (*n* = 4–5) taken from well-watered individuals (W), after withholding water for 1–27 days (d1–d27) and after re-watering for 1–5 days (r1–r5). The RWC of leaves collected from the filed at the peak of the dry season is also given (F, *n* = 9). Different letters above the bars indicate significant differences among means across treatment compared using Tukey’s honest significant difference test (*P* < 0.05).

**Figure S3.** Guttation (red circles) from leaf scars of *Paraboea rufescens* on a well-watered plant (A) and after re-watering plants from severe dehydration (B).

**Figure S4.** A typical leaf pressure–volume curve of *Paraboea rufecens*. The *x*-axis is relative water content (%), and *y*-axis is the inverse of leaf water potential (−MPa^−1^). The blue circle indicated the data points after turgor loss point and black circle indicated the data points before turgor loss point. The red line indicted the linear regression line for the data points after turgor loss point. The equation of the linear regression and the *R*^2^ were also shown. Turgor loss point leaf water potential (Ψ_tlp_, MPa) of this curve was −1.0 MPa, leaf relative water content at turgor loss point was 78 %. The osmotic potential at full turgor (π_100_, MPa) was −0.71 MPa, and the bulk modulus of elasticity along the entire range of positive turgor pressure (ε, MPa) was 2.8 MPa.

plac033_suppl_Supplementary_Material

## Data Availability

The data that support the findings of this study are available from the corresponding authors upon reasonable request.

## Notes

**Form & Function. Chief Editor**: Kate McCulloh
