# Supplementary material for: Leaf gas exchange and water relations of the woody desiccation-tolerant Paraboea rufescens during dehydration and rehydration
Source: AoB Plants. 2022 Jul 31;14(4):plac033. doi: 10.1093/aobpla/plac033 (PMC9403483; doi:10.1093/aobpla/plac033)
Supplement: plac033_suppl_Supplementary_Material [file plac033_suppl_supplementary_material.pdf]

SUPPORTING INFORMATION

Method S1 Data evaluation process of leaf pressure-volume curve

The leaf pressure-volume curve were analyzed with the Excel spreadsheet tool (https://prometheusprotocols.net/wp-content/uploads/2022/03/PVAST.xls) shared by Lawren Sack, Jessica Pasquet-Kok and Megan Bartlett (https://prometheusprotocols.net/function/water-relations/pressure-volume-curves/leaf-pressure-volume-curve-parameters/, access date April 17th, 2022). The process of the data analysis are as follows.

(1) Check for plateau effect and calculation of saturated leaf water content

The plateau effect caused by rehydration was firstly checked from the leaf water potential vs. leaf water content curve. The plateau effect exists if there is larger than expected initial decrease in leaf water content due to the excess apoplastic water (Kubiske and Abrams 1991). If plateau effect exists, then the data points at horizontal region of the leaf water content vs. leaf water potential curve would be taken out. Leaf saturated water content was calculated from leaf water vs. leaf water potential via linear regression of P-V data above and including the turgor loss point.

(2) Estimation of turgor loss point (Ψ_{tlp}, MPa), relative water content at turgor loss point, osmotic potential at full turgor (π_{100}, MPa) and bulk modulus of elasticity along the entire range of positive turgor pressure (ε, MPa).

Turgor loss point is the shifting point between curved and linear portion at the inverse leaf water potential vs. leaf relative water content. The position of change point needs to be adjusted to make the R^2 of the linear regression to be the highest and with at least five data points included. The leaf water potential at the shifting point is the turgor loss point leaf water potential, and the relative water content at turgor loss point was the relative water content at the shifting point. The inverse of the y-intercept of the linear regression was the osmotic potential at full turgor. The bulk modulus of elasticity along the entire range of positive turgor pressure were calculated from the slope of leaf water potential vs. leaf relative water content curve from the data points above and including turgor loss point.

Reference:

Kubiske ME, Abrams MD. 1991. Rehydration effects on pressure-volume relationships in four temperate woody species: variability with site, time of season and drought conditions. *Oecologia* 85: 537–542.

Sack L, Pasquet-Kok J, Bartlett M. Leaf pressure-volume curve parameters. Available at https://prometheusprotocols.net/function/water-relations/pressure-volume-curves/leaf-pressure-volume-curve-parameters/ Accessed April, 2022.

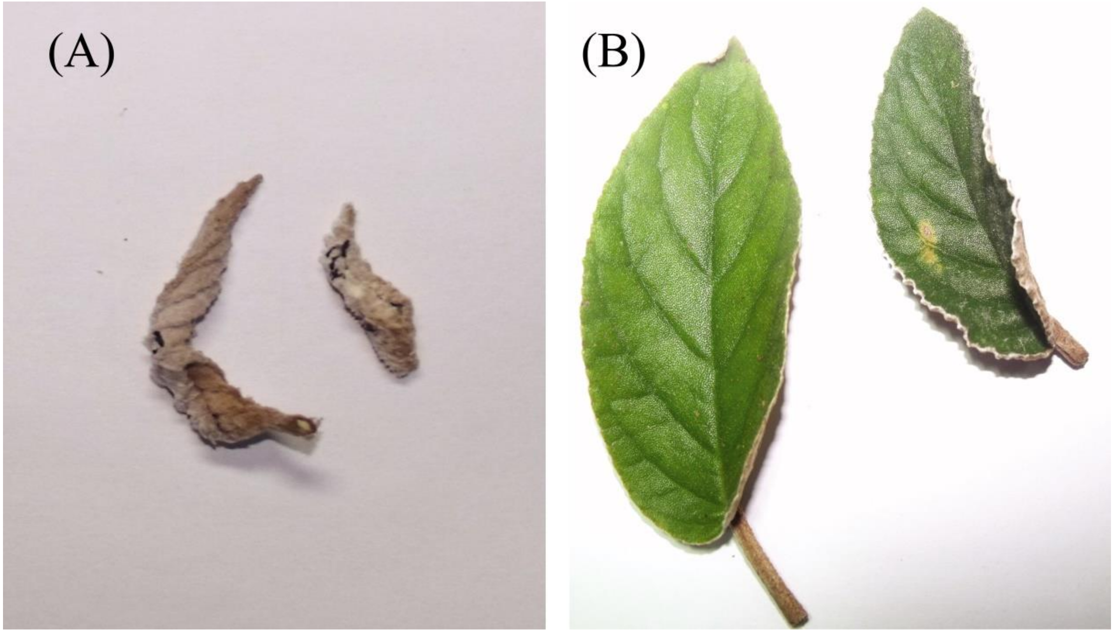

Fig. S1. Photographs of dehydrated *Paraboea rufescens* leaves collected from the field (A) and the same leaves after re-hydration on wet filter paper for 24 h (B).

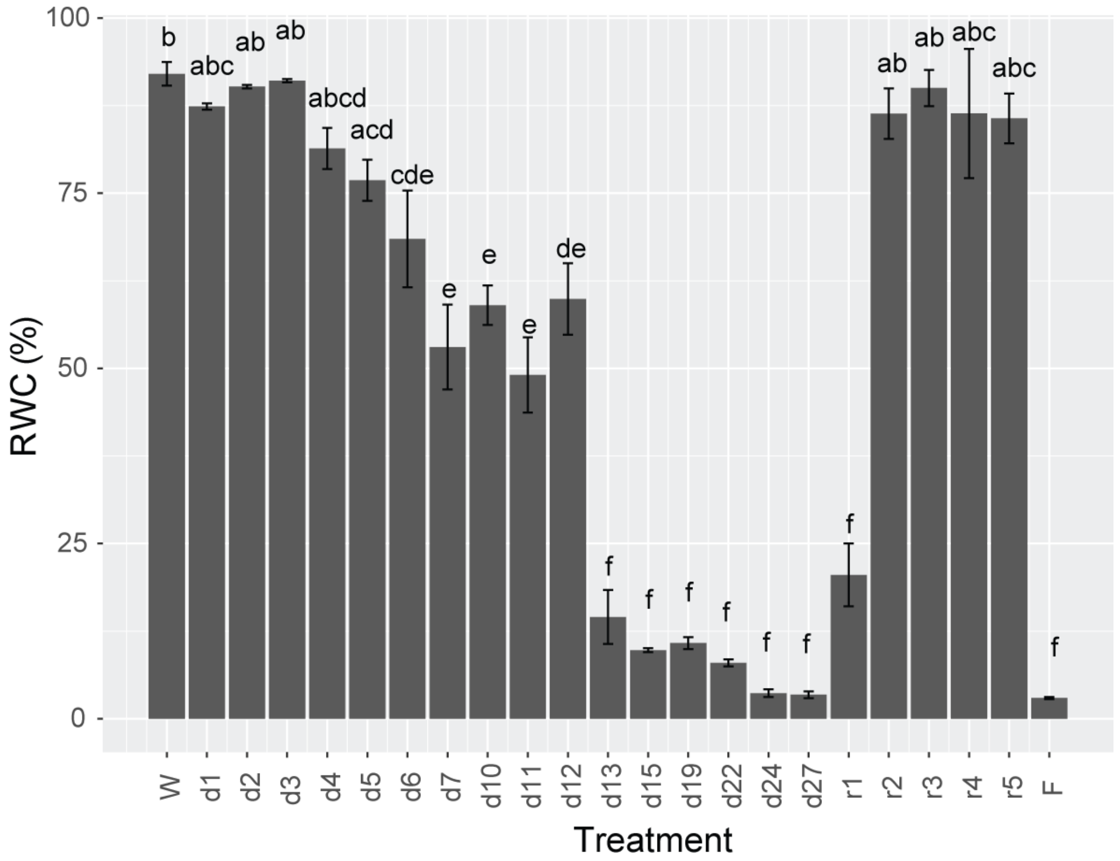

Fig. S2. Changes in leaf relative water content (RWC) in *Paraboea rufescens* during dehydration and re-watering. Values are means ± s.e. (n = 4-5) taken from well-watered individuals (W), after withholding water for 1 to 27 days (d1 to d27), and after re-watering for 1 to 5 days (r1 to r5). The RWC of leaves collected from the filed at the peak of the dry season is also given (F, n = 9). Different letters above the bars indicate significant differences among means across treatment compared using Tukey's honest significant difference test ($P < 0.05$).

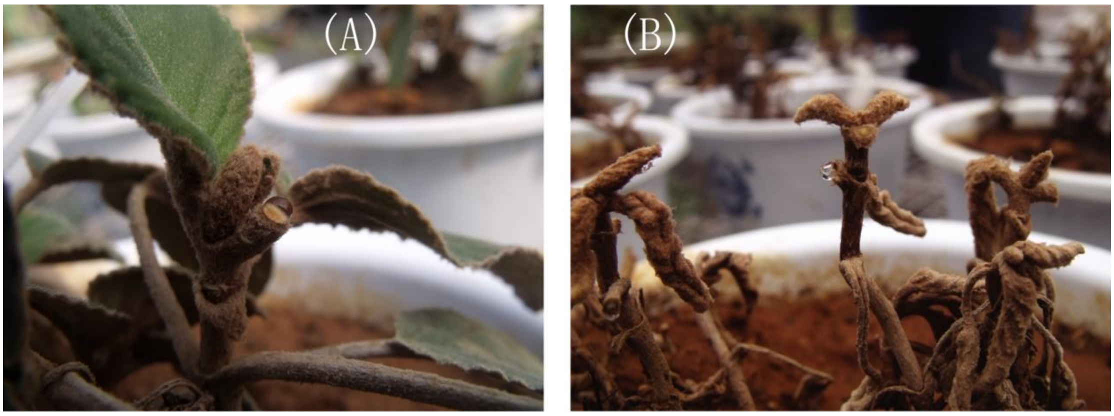

Fig. S3. Guttation (red circles) from leaf scars of *Paraboea rufescens* on a well-watered plant (A) and after re-watering plants from severe dehydration (B).

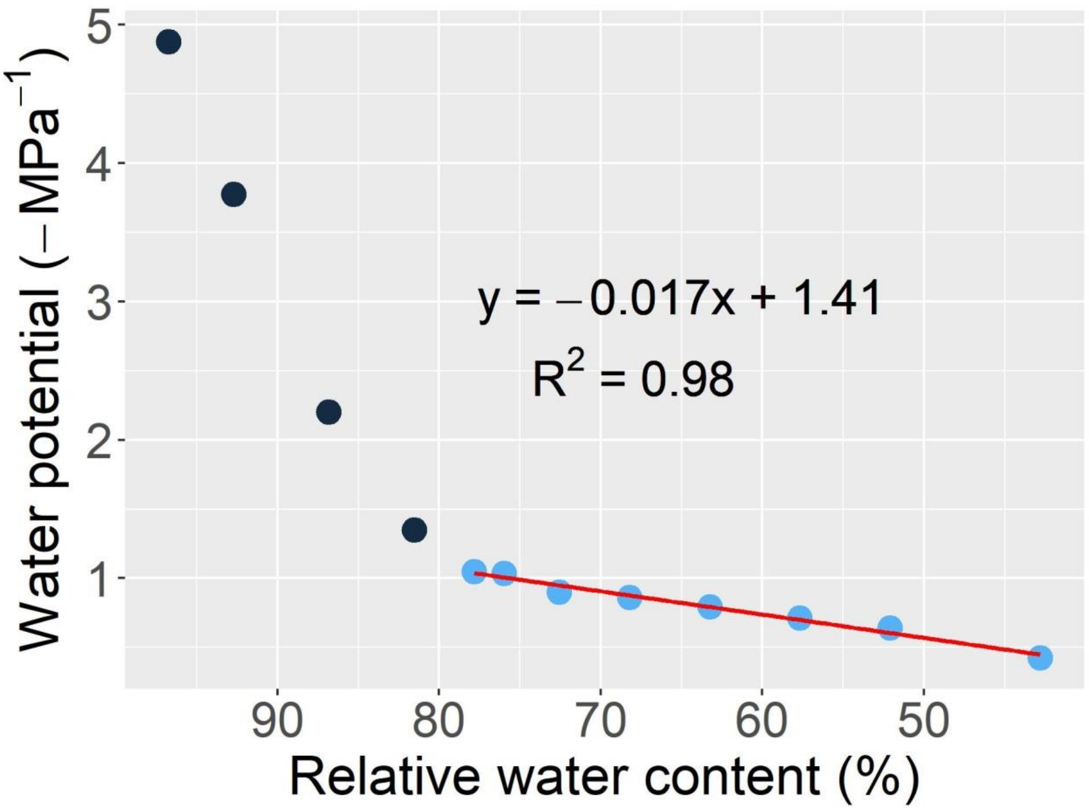

Fig. S4. A typical leaf pressure-volume curve of *Paraboea rufecens*. The x axis is relative water content (%), and y axis is the inverse of leaf water potential (-MPa^{-1}). The blue circle indicated the data points after turgor loss point and black circle indicated the data points before turgor loss point. The red line indicted the linear regression line for the data points after turgor loss point. The equation of the linear regression and the R^2 were also shown. Turgor loss point leaf water potential (Ψ_{tlp}, MPa) of this curve was -1.0 MPa, leaf relative water content at turgor loss point was 78%. The osmotic potential at full turgor (π_{100}, MPa) was-0.71MPa, and the bulk modulus of elasticity along the entire range of positive turgor pressure (ε, MPa) was 2.8 MPa.
